# Effect of Diamond-like Carbon Thin-Film Deposition on the Hardness of Pure Titanium Surfaces

**DOI:** 10.3390/ma18132992

**Published:** 2025-06-24

**Authors:** Hideaki Sato, Yutaka Kameyama, Ryota Yoshikawa, Kaito Tabuchi, Chizuko Ogata, Satoshi Komasa

**Affiliations:** 1 Department of Mechanical Engineering, Faculty of Science and Engineering, Tokyo City University, 1-28-1 Tamadutsumi, Setagaya-ku, Tokyo 158-8557, Japan; shsato@tcu.ac.jp (H.S.); ykameya@tcu.ac.jp (Y.K.); 2 Nomuraplating Co., Ltd., 5-12-20 Himejima, Nishiyodogawa-ku, Osaka 555-0033, Japan; r.yoshikawa@nomuraplating.co.jp; 3 Department of Oral Health Sciences, Faculty of Health Sciences, Osaka Dental University, 1-4-4, Makino-honmachi, Hirakata-shi 537-1144, Japan; tabuchi-k@cc.osaka-dent.ac.jp; 4 Department of Oral Health Engineering, Faculty of Health Sciences, Osaka Dental University, 1-4-4, Makino-honmachi, Hirakata-shi 537-1144, Japan; ogata-c@cc.osaka-dent.ac.jp

**Keywords:** oral implants, peri-implantitis, abutment, plaque removal, diamond-like carbon, pure titanium, surface roughness, curettage

## Abstract

The purpose of this study was to clarify the physical durability of a diamond-like carbon (DLC) thin film coated on pure titanium. The titanium surface of the abutment does not have sufficient toughness to prevent an increase in surface roughness or damage when the implant is scaled using a professional mechanical implement. The scaling process used for the removal of the dental plaque adhered to the abutment surface could increase the potential for the deposition of oral microorganisms and the accumulation of plaque, which increase the risk of peri-implantitis. A DLC thin film is biocompatible material that is known for its toughness, including extreme hardness, high abrasion resistance, chemical inertness, and high corrosion resistance. Protecting the abutment surface with the application of a DLC might prevent plaque adhesion due to its non-stick property. There was little change in the surface roughness of titanium samples to which DLC surface protection had been applied when the surface of the sample was scratched with a stainless steel scalar more than a thousand times. When cleaning the surface of pure titanium samples, the surface roughness significantly increased. DLC thin films are effective for the prevention the surface roughness of pure titanium implants from being increased when the conventional cleaning of the surface of the implant is performed.

## 1. Introduction

In recent years, implants have become essential as prosthetic treatments for missing teeth because of their predictability. However, with the widespread and continued advancement of dental implant technology, peri-implantitis has become an increasingly frequent issue in dental clinics [1,2,3]. Periodontopathic bacteria that infect the peri-implant area can cause peri-implantitis, an inflammatory lesion [4,5,6]. Similar to chronic periodontitis, the persistent infection symptoms include redness, peri-implant mucosal edema, and peri-implant bone loss. The implant bodies affected by peri-implantitis harbor bacteria commonly associated with chronic periodontitis [7,8,9,10]. The presence of oral bacteria at the time of implant insertion affects the biofilm production on the surface of the implant. As a result, bacteria from the periodontal pocket of the remaining teeth and subgingival plaque can serve as media for bacterial colonization on the newly placed implants [11,12,13,14].

Reversible peri-implant mucositis can rapidly progress to irreversible implant periodontitis, which destroys the osseointegration and bonding between the titanium implant material and bone tissue. This occurs because the adhesion between the implants and surrounding tissues differs significantly from that of natural teeth. For natural teeth, in addition to epithelial adhesion, there is connective tissue adhesion via fibers running directly to the cementum and root surface. These fibers function as the periodontal ligament and serve as a barrier against bacterial infection from the oral cavity [15,16,17,18,19,20]. In contrast, dental implants lack connective tissue adhesion to the titanium surface and lack the biological barrier function present in natural teeth. In addition, there is no active method of inhibiting bacterial adhesion to the exposed surfaces of the implant abutments in the oral cavity, and the insufficient strength of the abutment surface during plaque control prevents effective scaling and root planning, as performed for natural teeth.

The authors recognized the challenge of controlling infections through cleaning and developed a method to strengthen and modify the titanium surface of implants. In this study, we propose a surface modification technique for pure titanium that prevents the adhesion of contaminants to the implant abutment surface, prevents scratching of the implant abutment surface, and does not increase the surface roughness even after scaling and root planing. The core technology is using a coating of diamond-like carbon (DLC) on the abutment portion; DLC is a dry coating film consisting of an amorphous diamond-like material composed primarily of carbon. DLC is deposited to modify the surface of a material, providing wear resistance, low friction, and corrosion resistance [21,22,23,24]. It has a similar microstructure to diamond and possesses high hardness, heat resistance, and biocompatibility. In this study, DLC was deposited on the surface of pure titanium using a gas-cluster ion beam to investigate its wear resistance [25,26,27,28]. Since pure titanium is not used as a wear-resistant mechanical part, there are very few industrial examples of DLC coatings on the surface of pure titanium. However, this study investigated the modification of implant surfaces by depositing DLC on pure titanium implant abutments to examine the effectiveness of this method. To evaluate the effectiveness of the DLC coating on the implant surfaces, curettage was performed with a Gracey-type curette, which is commonly used on natural teeth

## 2. Materials and Methods

### 2.1. Sample Preparation

**Figure 1:**
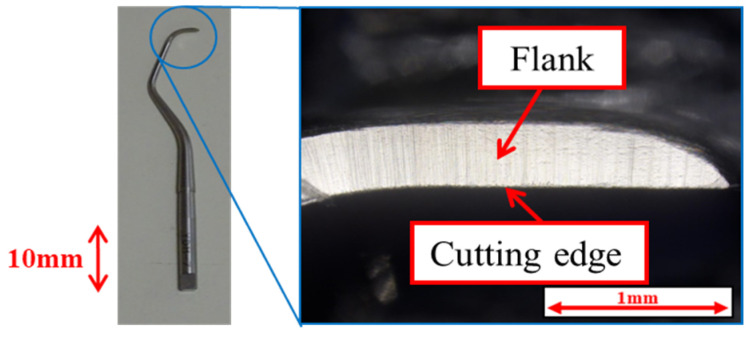
External view and enlarged view of the cutting edge of the Gracey-type curette.

**Figure 2:**
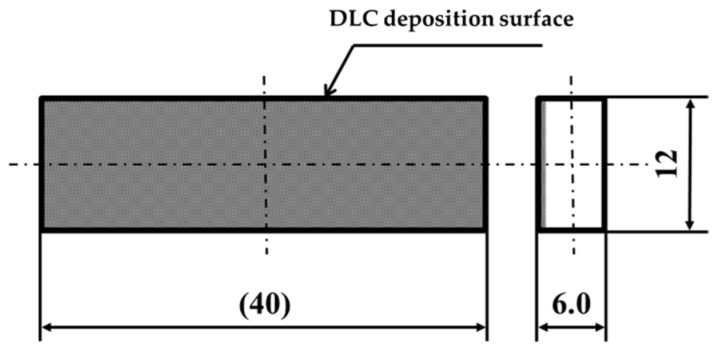
Shape of the pure titanium specimen.

This study used a stainless-steel scaler for natural teeth, specifically the Gracey-type curette, type #G7 manufactured by YDM Corporation (Tokyo, Japan). The material of the curette, equivalent to SUS440C, had a hardness of HRC88. Figure 1 provides an overall view and enlarged image of the edge of the scaler. In this study, the specimens shown in Figure 2 were fabricated using JIS Class 2 pure titanium (NIPPON STEEL CORPORATION, Tokyo, Japan). The specimens were ground on four surfaces using a surface grinding machine, and smooth surfaces with an arithmetic mean roughness Ra ≈ 0.10 µm were obtained using waterproof abrasive paper and a lapping sheet. DLC is a dry coating film that exhibits excellent properties such as high hardness, low friction, wear resistance, and corrosion resistance.

The DLC in this study was a thin film composed solely of carbon, with no hydrogen content (ta-C, tetrahedral amorphous carbon, tough carbon, Nomura Plating Co., Ltd., Osaka, Japan), providing extremely high structural stability and higher hardness (HV5 × 10^3^) compared with conventional DLC. A nanoindentation tester measured hardness. The film was deposited using a gas cluster ion beam (GCIB). The deposition conditions were a vacuum of 1 × 10^−5^ Pa, an acceleration voltage of 8.6 eV/atom, and 7.5 × 10^3^ Ar atoms/carbon atoms.

This study deposited DLC onto a smooth surface, which was then used as the test surface (Figure 2). Three types of DLC coatings were used: a 0.3 μm-thick film, a thicker 0.6 μm-thick film, and a 0.3 μm-thick film with a Cr intermediate layer (referred to as normal DLC 0.3 μm, normal DLC 0.6 μm, and Cr-DLC 0.3 μm, respectively). The thickness of the intermediate layer was 0.04 µm.

**Figure 3:**
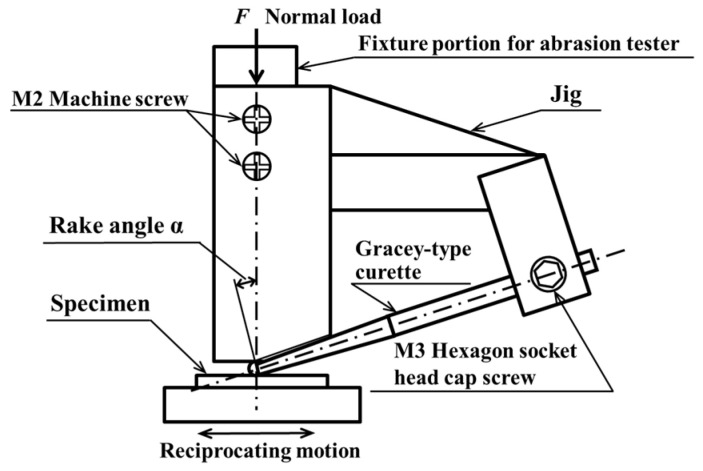
Gracey-type curette and jig.

During the experiment, a special jig was used to hold the scaler at a fixed angle. The jig was fabricated by cutting and welding stainless steel (SUS304). The scaler was fixed to the jig using screws. A stainless-steel post was attached to the jig to prevent the scaler from deflection due to movements. Figure 3 shows a diagram of the scaler attached to the jig.

### 2.2. Lifetime Tests

A special type of plane abrasion tester (manufactured by Toyo Seiki Seisaku-sho, Ltd. Tokyo, Japan) was used to test the life of the DLC deposited on pure titanium. The test specimen was fixed to a reciprocating table using a jig, and a Gracey-type curette was attached to the fixture. The experimental conditions of the life tests are listed in Table 1. The number of curettages N was based on 3 × 10^3^ curettage cycles, which was calculated based on the guidelines of the Japanese Society of Oral Implantology and The Japanese Society of Periodontology (assuming a patient who needs treatment once a month, the number of curettage cycles per cleaning was considered to be 25, 300 times per year, and 3 × 10^3^ times in 10 years), and the number of curettage cycles was increased, with the number of curettages set to N = 4 × 10^3^ and 1 × 10^4^ times. The Ra of the four groups was compared using Mann–Whitney U tests using a significance level of 0.05. Data analysis was conducted using EXCEL TOKEI v.8.0 (ESUMI Co., Ltd., Tokyo, Japan).

### 2.3. Methods of Evaluating Lifetime Test Surfaces

The test surfaces were evaluated by magnifying them using an optical microscope, cross-sectional curves, and the arithmetic mean roughness Ra. From the magnified observation of the test surface and cross-sectional curve, the surface damage caused by the curettage test was confirmed, and the arithmetic mean roughness of the surface was used to evaluate whether the surface after the test was suitable for use in the oral cavity. The initial surface roughness was set to Ra ≤ 0.2 µm. This is the surface roughness recommended by Bollen et al. An optical microscope (VHX-1000, Keyence Corporation, Osaka, Japan) was used to measure the cross-sectional curves, and a SURFCOM FLEX-50A stylus-type surface roughness meter (TOKYO SEIMITSU Co., Ltd., Tokyo, Japan) was used to measure the surface roughness. Surface roughness was measured at 10 locations perpendicular to the reciprocating direction of the curettage, and the mean value and standard deviation were calculated. The Ra of the four groups was compared using Mann–Whitney U tests using a significance level of 0.05. Data analysis was conducted using EXCEL TOKEI v.8.0 (ESUMI Co., Ltd., Tokyo, Japan).

### 2.4. Wet Test Assuming Oral Cavity

In clinical practice, curettage is performed in a wet oral cavity. Therefore, it was necessary to conduct curettage tests of the DLC in a wet environment to evaluate the effectiveness of the DLC coatings on dental implants. The DLC surface was kept wet in this study, and a wet curettage test was conducted. The same special type of plane abrasion tester (manufactured by Toyo Seiki Seisaku-sho, Ltd. Tokyo, Japan) used for the life test was used as the testing machine. The wet test conditions are presented in Table 2. After the test, the surface roughness was measured using an optical microscope and a stylus-type surface roughness meter. The Ra of the four groups was compared using Mann–Whitney U tests using a significance level of 0.05. Data analysis was conducted using EXCEL TOKEI v.8.0 1(ESUMI Co., Ltd., Tokyo, Japan).

### 2.5. Measurement of Coefficient of Friction and Observation of Curettage Marks

**Figure 4:**
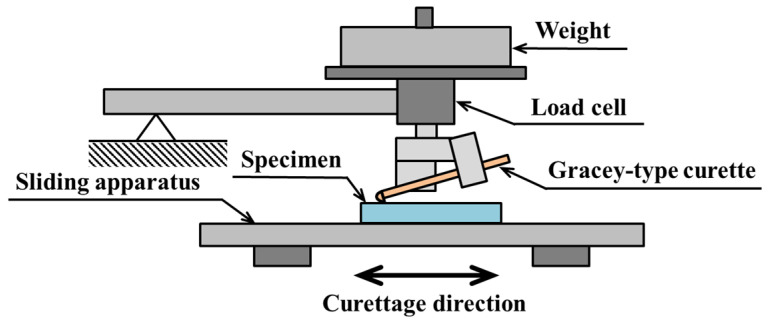
Experimental apparatus.

The coefficient of friction was measured using a reciprocating sliding-type wear friction tester, a Tribogear TYPE 38 (Shinto Scientific Co., Ltd., Tokyo, Japan). Figure 4 shows a schematic diagram of the experimental apparatus.

The scaler was attached to the surface of the specimen by the jig described above. By applying a weight to the jig, the scaler exerted a force on the surface of the specimen. During the test, the table was moved back and forth horizontally, as indicated by the arrow, while the scaler and specimen were in contact. A load cell was attached to the part holding the jig, the friction force was created by sliding, and the coefficient of friction was calculated. The test conditions are shown in Table 3.

## 3. Results

### 3.1. Lifetime Tests

**Figure 5:**
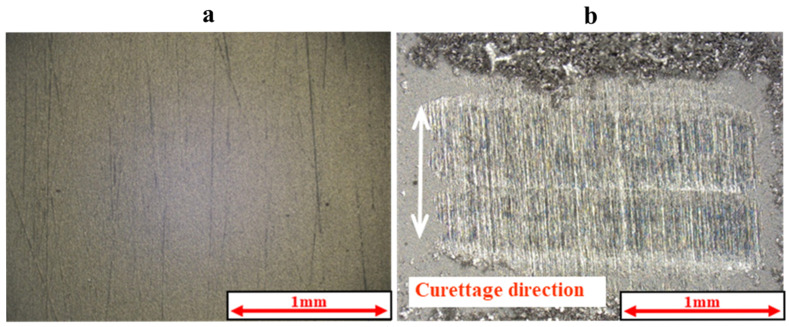
The surface of JIS Class 2 pure titanium specimen before and after curettage (N = 1 × 10^3^, F = 3.9 N) ((**a**) before curettage, (**b**) after curettage).

**Figure 6:**
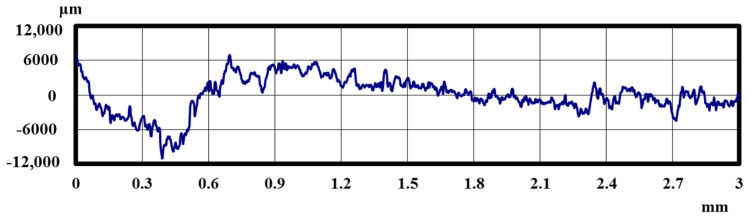
The cross-sectional curve of JIS Class 2 pure titanium specimen after curettage (N = 1 × 10^3^, F = 3.9 N).

**Figure 7:**
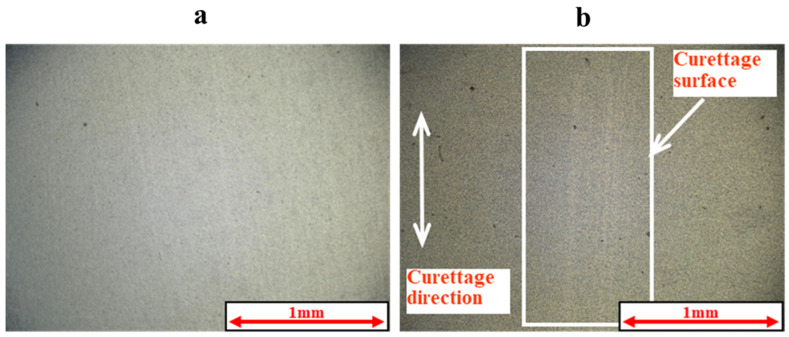
The surface of normal DLC 0.3 µm deposited on JIS Class 2 pure titanium specimen before and after curettage (N = 3 × 10^3^, F = 3.9 N) ((**a**) before curettage, (**b**) after curettage).

**Figure 8:**
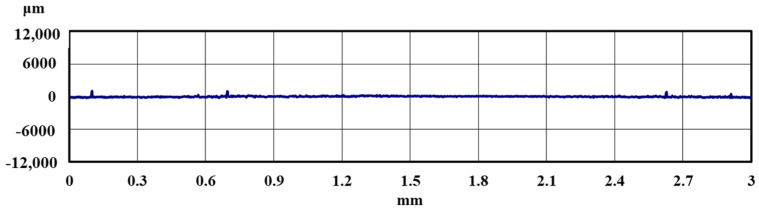
The cross-sectional curve of normal DLC 0.3 µm deposited on JIS Class 2 pure titanium specimen after curettage (N = 3 × 10^3^, F = 3.9 N).

Figure 5 and Figure 6 show the results of the magnified observation of the specimen and cross-sectional curves after curettage using a Gracey-type stainless-steel curette on pure titanium without DLC coating. Figure 7 and Figure 8 show the results on pure titanium with a normal DLC of 0.3 μm. The number of curettages was 3 × 10^3^ times.

As shown in Figure 5 and Figure 6, the surface was significantly damaged when the surface of pure titanium without DLC was subjected to curettage with a stainless-steel curette. On the other hand, no significant damage was observed on the surface of pure titanium with normal 0.3 μm DLC deposition, as shown in Figure 7 and Figure 8 for before and after curettage, and the formation of curettage marks was suppressed.

**Figure 9:**
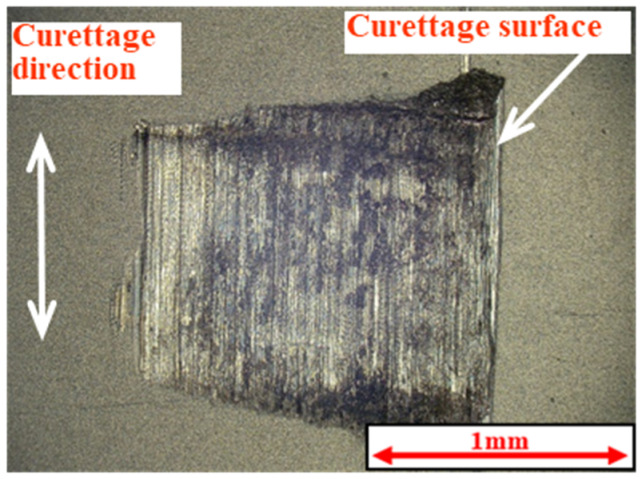
The surface of normal DLC 0.3 µm deposited on JIS Class 2 pure titanium specimen after curettage (N = 4 × 10^3^, F = 3.9 N).

**Figure 10:**
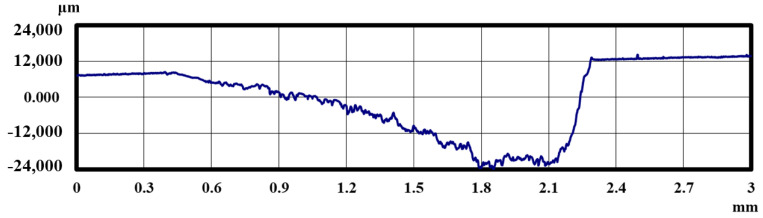
The cross-sectional curve of normal DLC 0.3 µm deposited on JIS Class 2 pure titanium specimen after curettage (N = 4 × 10^3^, F = 3.9 N).

**Figure 11:**
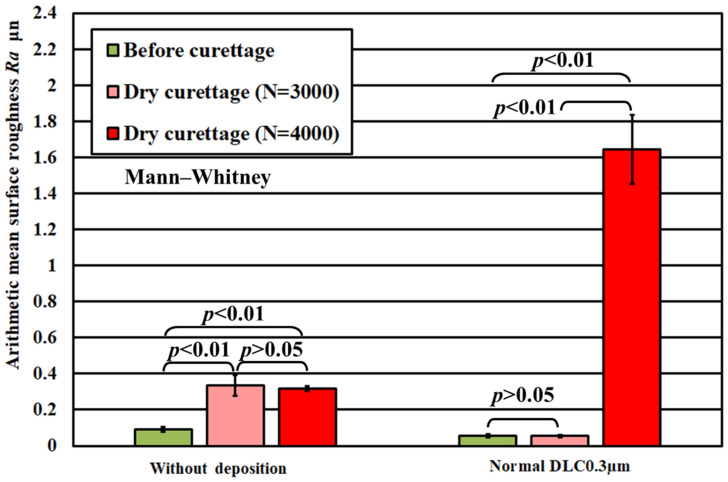
Arithmetic mean surface roughness of the specimen surface before and after curettage (F = 3.9 N).

These results confirmed the wear resistance of the DLC film to curettage. Next, Figure 9 and Figure 10 show the results of the magnified observation of the specimen surface after curettage and the cross-sectional curves of pure titanium on which normal DLC 0.3 μm was deposited when the number of curettages increased to 4 × 10^3^ times. Figure 11 shows the relationship between the number of curettage cycles and the arithmetic mean surface roughness Ra.

The wear resistance of normal 0.3 μm DLC was confirmed up to 3 × 10^3^ curettage cycles, but, as shown in Figure 9 and Figure 10, the DLC peeled off when the number of curettages increased to 4 × 10^3^ times, and the pure titanium base material was cut away.

Figure 11 shows that the arithmetic mean surface roughness Ra of the pure titanium coated with a normal DLC 0.3 µm had a surface roughness Ra ≦ 0.2 µm after 3 × 10^3^ curettage cycles. Still, when peel-off was confirmed, the arithmetic mean surface roughness Ra increased approximately 5.2 times compared to the value after curettage under the same conditions on pure titanium with no deposition. The condition of Ra ≦ 0.2 µm was not satisfied. Based on these results, it is necessary to have sufficiently durable deposition conditions to prevent peel-off as much as possible when applying DLC coatings. Therefore, for improving the durability of DLC, two new types of DLC were used: (1) normal DLC 0.6 μm, with a DLC film thickness of 0.6 μm and (2) DLC 0.3 μm using Cr as an intermediate layer with the same film thickness, and their wear resistance to curettage was evaluated.

**Figure 12:**
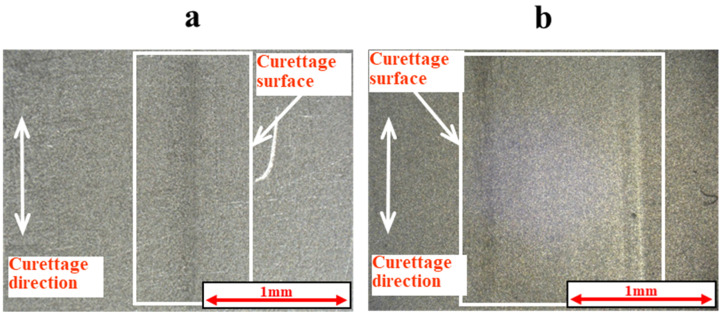
The surface of normal DLC 0.6 µm deposited on JIS Class 2 pure titanium specimen after curettage (F = 3.9 N) ((**a**) N = 4 × 10^3^, (**b**) N = 1 × 10^4^).

**Figure 13:**
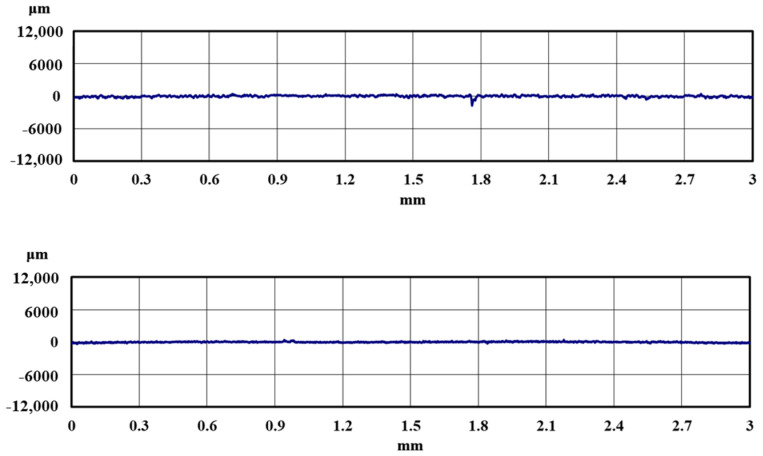
The cross-sectional curve of normal DLC 0.6 µm deposited on JIS Class 2 pure titanium specimen after curettage (F = 3.9 N). (**upper**: N = 4 × 10^3^, **lower**: N = 1 × 10^4^).

**Figure 14:**
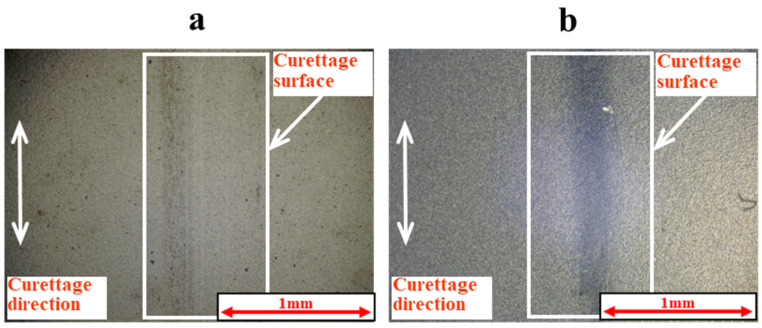
The surface of DLC 0.3 µm with Cr intermediate layer deposited on JIS Class 2 pure titanium specimen after curettage (F = 3.9 N) ((**a**) N = 4 × 10^3^, (**b**) N = 1 × 10^4^).

**Figure 15:**
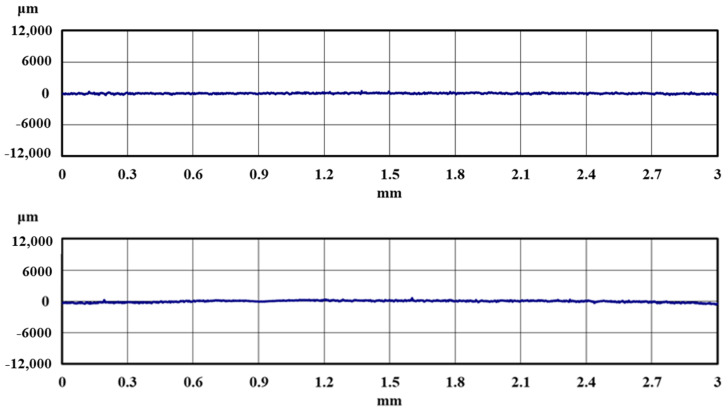
The cross-sectional curve of DLC 0.3 µm with Cr intermediate layer deposited on JIS Class 2 pure titanium specimen after curettage (F = 3.9 N). (upper: N = 4 × 10^3^, lower: N = 1 × 10^4^).

**Figure 16:**
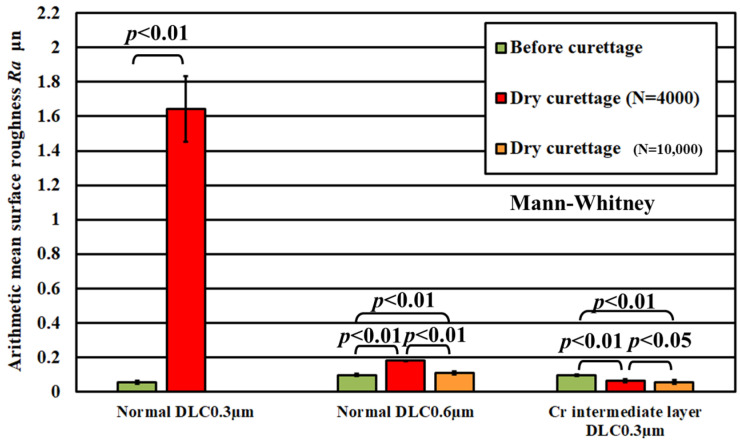
Arithmetic mean surface roughness of the specimens’ surfaces before and after curettage for three types of DLC (F = 3.9 N).

**Figure 17:**
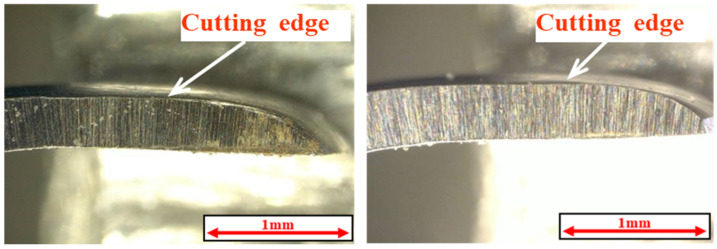
Enlarged view of the cutting edge of the Gracey-type curette used for the curettage (N = 1 × 10^4^, F = 3.9 N) (left: normal DLC 0.6 µm, right: DLC 0.3 µm with Cr intermediate layer).

Figure 12 and Figure 13 show the observation results of a specimen of pure titanium on which normal DLC 0.6 μm was deposited after 4 × 10^3^ and 1 × 10^4^ curettage cycles. Figure 14 and Figure 15 show the observation results of a specimen of pure titanium on which a DLC film 0.3 μm thick with a Cr intermediate layer was deposited after 4 × 10^3^ and 1 × 10^4^ curettage cycles. Figure 12 and Figure 13 show that when the DLC thickness increased, no peeling of the DLC occurred even after 4 × 10^3^ cycles of curettage. Also, even when the number of curettages was significantly increased to 1 × 10^4^ times, no exfoliation occurred, and the surface roughness also met the condition of Ra ≦ 0.2 µm. Therefore, it was considered that normal DLC 0.6 µm had sufficient durability against the number of curettages. Similarly, DLC with Cr added as the intermediate layer, shown in Figure 14 and Figure 15, was tested for 4 × 10^3^ and 1 × 10^4^ cycles, respectively, but no peeling of the DLC occurred. Figure 16 shows the surface roughness, and Figure 17 shows the results of the magnified observation of the edge of the scaler used for the N = 1 × 10^3^ cycle test. Since no significant wear was observed on the edge ridge of the scaler used for the normal DLC 0.6 μm, as shown in Figure 17, it was considered that the black wear powder was not caused by the wear of the stainless-steel scaler, but that the scaler’s scraping curetted off the droplets on the DLC surface.

### 3.2. Lifetime Test Results by Wetting, Assuming an Oral Cavity

**Figure 18:**
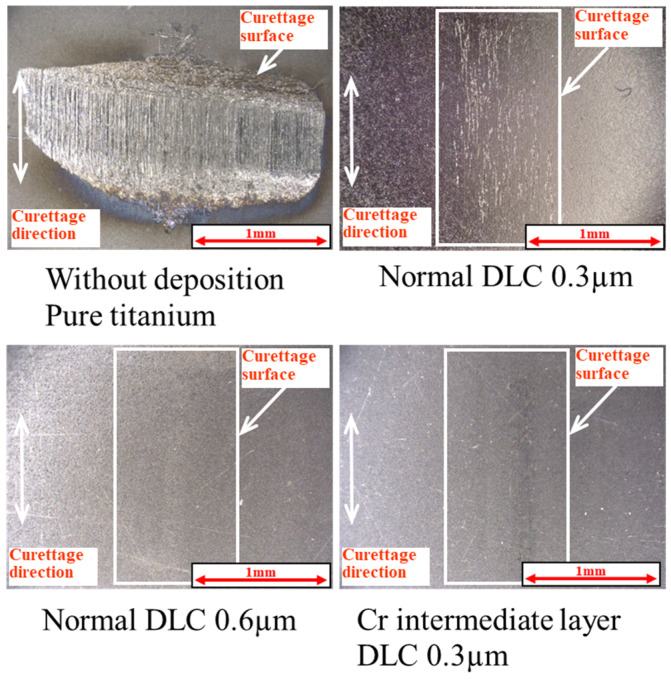
The surface of the specimen after wet curettage (N = 1 × 10^4^, F = 3.9 N).

**Figure 19:**
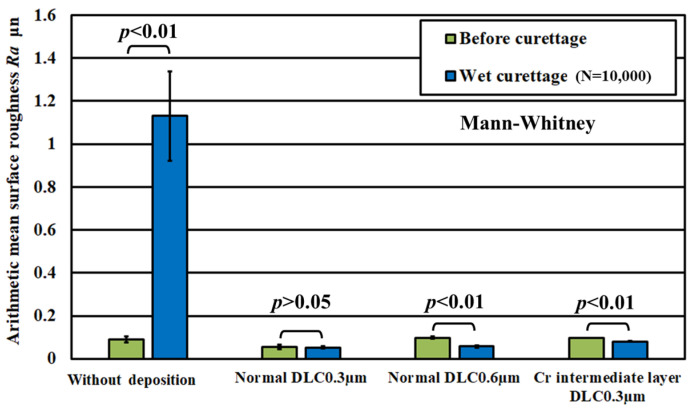
Arithmetic mean surface roughness of the specimen surface before and after wet curettage (N = 1 × 10^4^, F = 3.9 N).

Figure 18 shows the results of the magnified observation of the test surface after wet curettage, assuming an intraoral cavity, and Figure 19 shows the surface roughness of the specimen. The curettage marks formed on the surface were smaller than those observed after dry curettage.

**Figure 20:**
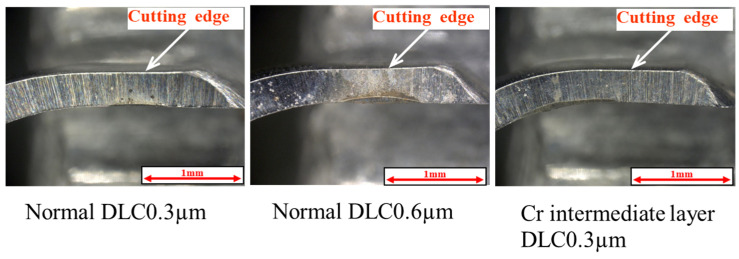
Enlarged view of the cutting edge of the Gracey-type curette used for the wet curettage (N = 1 × 10^4^, F = 3.9 N).

Figure 19 shows that the surface roughness of the pure titanium coated with the three types of DLC satisfied the condition of Ra ≦ 0.2 µm even after N = 1 × 10^4^ curettage cycles. From this result, it can be inferred that performing curettage in a wet environment effectively improves the durability of DLC deposited on pure titanium. Figure 20 shows the results of the magnified observation of the cutting edges of the scaler used in the wet test, where peeling and curettage scar formation on the DLC surface were suppressed in the wet test.

### 3.3. Coefficient of Friction Measurement Test Results

**Figure 21:**
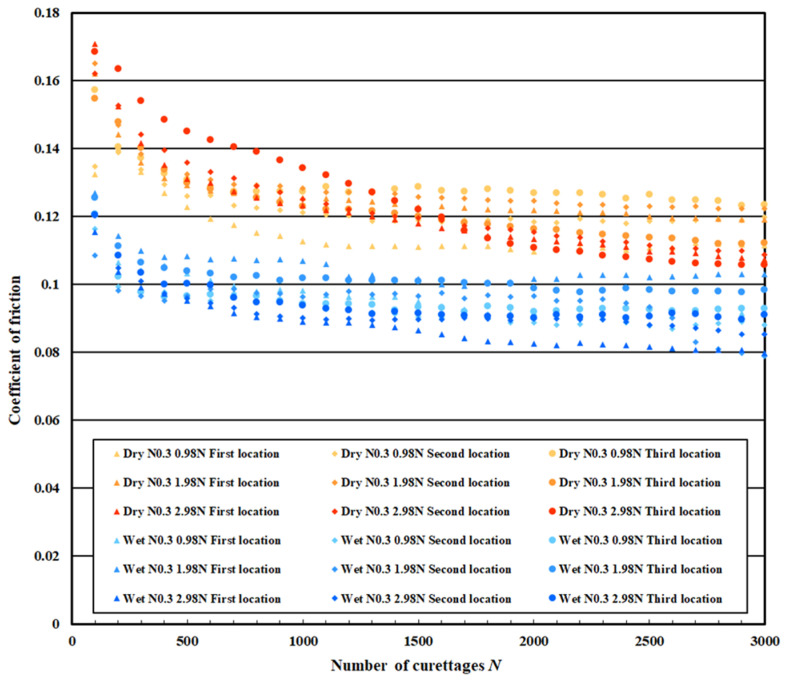
The coefficient of friction of normal DLC 0.3 µm.

**Figure 22:**
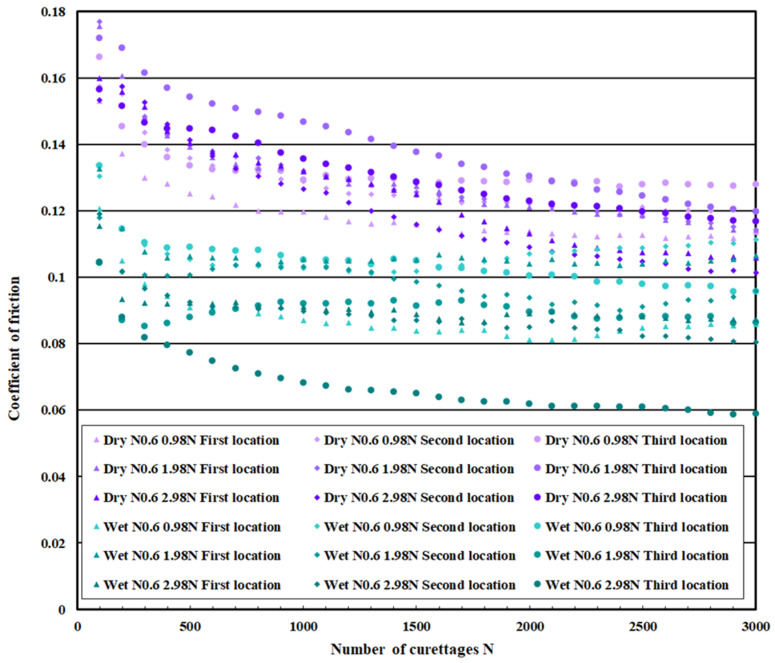
The coefficient of friction of normal DLC 0.6 µm.

**Figure 23:**
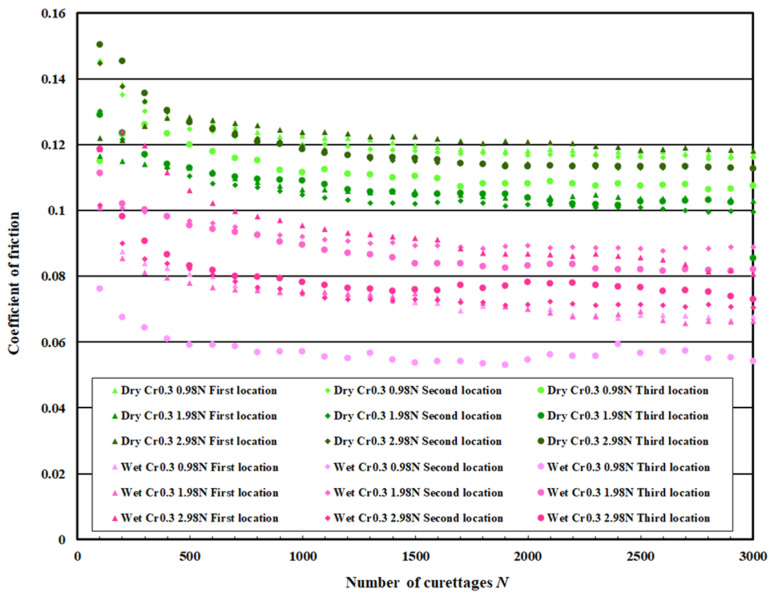
The coefficient of friction of DLC 0.3 µm with Cr intermediate layer.

Figure 21 shows the measurement results of the coefficient of friction on the pure titanium surface on which 0.3 μm normal DLC was deposited, Figure 22 for 0.6 μm normal DLC, and Figure 23 for 0.3 μm DLC with a Cr intermediate layer. Table 4 lists the average values calculated for the initial friction range (N = 0–100 times) and after N = 2500, when the coefficient of friction was stable. In both cases, the coefficient of friction in the wet test was lower than that in the dry test.

This coefficient of friction indicates that the DLC improved durability under wet conditions. Comparing the coefficient of friction of each DLC, no significant difference was observed, but the coefficient of friction of DLC 0.3 μm with the Cr intermediate layer was slightly decreased. There was no significant increase or decrease in the coefficient of friction for the regular DLC 0.6 μm, and the values varied.

## 4. Discussion

Reversible peri-implant mucositis rapidly progresses to irreversible implant periodontitis, which destroys the bond between the implant material, titanium, and bone tissue. This is because the adhesion between implants and the surrounding tissues differs significantly from that of natural teeth. Dental implants do not have connective tissue adhering to the titanium surface and are considered poor biological barriers against infection compared to natural teeth. In addition, there is no active method for inhibiting bacterial adhesion to the abutment surface exposed in the oral cavity, and the lack of strength of the abutment surface during plaque control makes it impossible to clean the surface as thoroughly as natural teeth. The authors believe it is important to address the areas where cleaning is difficult by applying infection control to prevent peri-implantitis and devise a new method to deal with this problem by strengthening or modifying the titanium surface, which is used as the implant material [1,2,3,4,5,6,7,8,9,10,11,12,13,14].

This study deposited DLC on a pure titanium surface, and curettage experiments were conducted using a stainless-steel scaler to evaluate the surface properties. The following findings were obtained: When a stainless-steel scaler was used for the curettage of the pure titanium surface, the surface was severely damaged; however, the damage was suppressed by DLC deposition on the pure titanium surface, and the effect of DLC deposition on the resistance to wear and tear against curing was confirmed. When the DLC peeled off, the surface roughness increased significantly and became higher than pure titanium without DLC. Therefore, it is considered that a DLC that does not peel off during scraping or during curettage is necessary. A comparison of the two improved coatings, by increasing the thickness of the DLC film and adding an intermediate layer of Cr, shows that using an intermediate layer of Cr is more suitable for improving implant lifetime in terms of wear particles, changes in surface roughness, and friction coefficient.

The DLC used in this study was a thin film composed only of carbon and did not contain hydrogen. Its structure was amorphous and non-crystalline, and it had many superior properties compared with conventional hydrogen-containing DLC. Its hardness was 5 × 10^3^ HV Vickers, two to three times harder than conventional films. The formation temperature can be kept below 100 °C by optimizing the interface treatment between the base material and tough carbon, and the stress at the film interface can be alleviated by reducing the formation temperature, resulting in high adhesion [21,22,23,24].

In this study, as a preparatory step for the clinical application of DLC on the surface of implant abutments, a Gracey-type curette scaler for natural teeth was fixed in a dedicated jig and used to curette pure titanium without DLC coating. The effects of applying a constant load using the scaler on the properties and durability of the pure titanium surface was evaluated. The scratch marks formed on the surface of the JIS Class 2 pure titanium specimen were observed by magnifying the test surface and creating a profile curve.

A normal DLC 0.3 µm specimen was curetted 3 × 10^3^ times with a stainless-steel scaler, and the surface damage was suppressed, satisfying the surface roughness requirement of Ra ≦ 0.2 µm, which is the condition for suppressing plaque adhesion.

This suggests that DLC is effective in improving the wear resistance of implant abutments against scaling and root planing, which was the main objective of this study. However, as the number of curettages increased, the results of macroscopic observation and profile curves showed that the normal DLC 0.3 μm peeled off from the specimen causing significant damage to the base material, the pure titanium, and increasing the surface roughness compared to the specimens without DLC. Therefore, it is necessary to find conditions under which the DLC applied to the pure titanium peels off as little as possible.

In this study, a test was conducted to increase the number of curettages by increasing the thickness of the DLC. As a result, it was confirmed that the surface roughness of the specimen with a film thickness of 0.6 μm was Ra ≦ 0.2 µm, and the DLC did not peel off, even when the number of curettages was increased. This indicates that increasing the thickness of the DLC can withstand long-term scraping. On the other hand, when the surface of normal DLC with a thickness of 0.6 μm was observed under magnification, it was confirmed that black wear powder was generated as the number of curettages increased.

As no increase in wear was observed on the cutting edge, which is the contact area, from the results of the observation of the cutting edge of the scaler used, it was possible that the wear powder observed from the magnified observation of the specimen surface was caused by the graphite-like transformation of the DLC owing to friction with the scaler, resulting in wear [16]. The surface roughness measurement results showed a decreased surface roughness with an increase in the number of curettages, and the coefficient of friction showed a transition in the coefficient of friction. These results also suggest that increased internal stress due to increased DLC film thickness may be involved and that some compositional change may have occurred on the DLC surface during scraping and curettage by the scaler [29,30].

The effect of the DLC coating on the wear resistance of the pure titanium specimens against scaling was confirmed. By increasing the DLC film thickness, the specimens could be used for a more extended period than expected. However, the observed wear debris and the changes in the surface properties indicate that the DLC surface is easily worn away, and increasing the film thickness does not solve the problem of insufficient wear resistance against scaling. Therefore, scaling tests were performed on the specimens using a flat abrasion tester in which a Cr intermediate layer was inserted during the DLC film deposition process, and the effect of the Cr intermediate layer on the wear resistance of DLC was examined [31,32,33].

The DLC 0.3 μm specimen with a Cr intermediate layer inserted during DLC deposition was as thick as the normal DLC 0.6 μm specimen. It met the condition of Ra ≦ 0.2 µm, which suppresses surface damage, increases surface roughness, and suppresses plaque adhesion, even after long-term scratching that was significantly longer than expected in clinical settings. Therefore, inserting a Cr intermediate layer is considered a suitable means of improving the wear resistance of DLC coatings on pure titanium in dental implants.

The magnified observation of the specimen surface and the scaler showed no wear debris, similar to that of the normal DLC 0.6 μm specimen, and the friction coefficient measurement showed a slight decrease in the friction coefficient. The surface roughness measurements showed almost no change in surface roughness with an increasing number of curettages. The DLC 0.3 μm specimen with a Cr intermediate layer is considered to be more effective than the normal DLC 0.6 μm specimen.

Although dry tests were conducted, the oral cavity, in which dental implants are clinically used, is a wet environment. Three types of DLC specimens, normal DLC 0.3 μm, normal DLC 0.6 μm, and DLC with a Cr interlayer 0.3 μm or thicker, were subjected to lifetime and wear friction tests in a wet environment to investigate the effects on wear resistance. The wet curettage, which was performed on each specimen under the assumption that it was in the oral cavity, confirmed that the pure titanium specimens without a DLC coating showed significant damage and increased surface roughness due to scaling, indicating that it is difficult to curettage them in the same manner as natural teeth, even in a wet environment.

On the other hand, the three DLC-coated specimens, including the normal DLC 0.3 μm specimen on which DLC exfoliation was confirmed in the 4 × 10^3^ cycles of dry curettage, showed no evidence of DLC exfoliation or increased surface roughness even after 1 × 10^4^ cycles of wet curettage, which was well beyond the 3 × 10^3^ cycles of curettage that were assumed in this study for clinical use. The surface was not rough even after 1 × 10^4^ cycles of curettage, which is well over the 3 × 10^3^ cycles of curettage assumed in this study. Therefore, a wet environment was considered to have no adverse effect on the wear resistance of DLC deposited on pure titanium when the DLC was subjected to curettage with a scaler.

A comparison of the coefficient of friction measurement results for each DLC specimen showed no significant difference. Still, the coefficient of friction of the DLC 0.3 μm specimen with a Cr interlayer was slightly lower than that of the DLC 0.3 μm specimen. The coefficient of friction measurements of the DLC specimens showed that the coefficient of friction during wet curettage was lower than that during dry curettage. However, observation of the cutting edge, which is the contact area of the scaler with the specimen, showed that it was significantly worn.

This is because the contact area between the scaler and test surface was always wet because of the wet test, and the Cr contained in the stainless steel, which had a lower hardness than the DLC, was not exposed to the atmosphere when it was worn away owing to sliding. This is because iron, the main component of stainless steel, corroded and embrittled due to the inhibition of passive film formation.

## 5. Conclusions

In this study, DLC was deposited on a pure titanium plane to improve the wear resistance of the abutment surface of dental implants. Curettage experiments were conducted to evaluate the surface properties, assuming scaling and root planing using a stainless-steel scaler, a standard plaque control method for natural teeth. The surface properties were evaluated. The scaling and root planing of a pure titanium surface without the DLC coating were conducted using a stainless-steel scaler, similar to that used for natural teeth, which resulted in surface damage and increased surface roughness, adversely affecting the material. The improvement in wear resistance against scraping and root planing using a stainless-steel scaler was confirmed by depositing DLC on a pure titanium surface. In addition, when the peeling off the deposited DLC was assumed, a comparison of two improvement methods, increasing the thickness of the DLC and inserting a Cr intermediate layer, showed that both were effective in improving the service life with increases in the number of curettages and load. However, from the viewpoint of the degree of surface damage and the change in the coefficient of friction with increasing load, the insertion of a Cr intermediate layer is considered more suitable for improving service life.

## Figures and Tables

**Table 1:** Lifetime test experimental conditions.

Specimen	Without deposition, pure titanium Normal DLC 0.3 μm Normal DLC 0.6 μm Cr intermediate layer DLC 0.3 μm
Scaler	Gracey-type curette type #G7
Normal force, *F* N	3.9
Rake angle, *α* degree	10
Number of curettages, *N* times	1 × 10^3^, 3 × 10^3^, 4 × 10^3^, 1 × 10^4^
Curettage length, *L* mm	3
Curettage direction	Reciprocating motion

**Table 2:** Wet test experimental conditions.

Specimen	Without deposition, pure titanium Normal DLC 0.3 μm Normal DLC 0.6 μm Cr intermediate layer DLC 0.3 μm
Scaler	Gracey-type curette type #G7
Normal force, *F* N	3.9
Rake angle, *α* degree	10
Number of curettages, *N* times	1 × 10^4^
Curettage length, *L* mm	3
Flow rate, m^3^/s	4 × 10^−6^
Curettage direction	Reciprocating motion

**Table 3:** Experimental conditions for coefficient of friction.

Specimen	Without deposition, pure titanium Normal DLC 0.3 μm Normal DLC 0.6 μm Cr intermediate layer DLC 0.3 μm
Scaler	Gracey-type curette type #G7
Normal force, *F* N	0.98, 1.98, 2.98
Rake angle, *α* degree	10
Number of curettages, *N* times	3 × 10^3^
Curettage length, *L* mm	3
Curettage fluid	Dry Wet (curettage was performed while water was dripped onto the specimen surface)
Curettage direction	Reciprocating motion

**Table 4:** Coefficient of friction results.

Normal DLC 0.3 μm	Dry curettage			Wet curettage		
	*F* = 0.98 N	*F* = 1.98 N	*F* = 2.98 N	*F* = 0.98 N	*F* = 1.98 N	*F* = 2.98 N
*N* = 0~100	0.14	0.16	0.17	0.12	0.12	0.12
*N* = 2.5~3 × 10^3^	0.12	0.12	0.11	0.10	0.110	0.09
Normal DLC 0.6 μm	Dry curettage			Wet curettage		
	*F* = 0.98 N	*F* = 1.98 N	*F* = 2.98 N	*F* = 0.98 N	*F* = 1.98 N	*F* = 2.98 N
*N* = 0~100	0.16	0.18	0.16	0.13	0.12	0.11
*N* = 2.5~3 × 10^3^	0.12	0.12	0.11	0.10	0.10	0.08
DLC 0.3 μm with Cr intermediate layer	Dry curettage			Wet curettage		
	*F* = 0.98 N	*F* = 1.98 N	*F* = 2.98 N	*F* = 0.98 N	*F* = 1.98 N	*F* = 2.98 N
*N* = 0~100	0.14	0.13	0.14	0.09	0.11	0.11
*N* = 2.5~3 × 10^3^	0.11	0.10	0.12	0.06	0.08	0.08

## Data Availability

The original contributions presented in this study are included in the article. Further inquiries can be directed to the corresponding author.
